# Comprehensive Effects of Near-Infrared Multifunctional Liposomes on Cancer Cells

**DOI:** 10.3390/molecules25051098

**Published:** 2020-03-01

**Authors:** Yiqing Deng, Huaying Huang, Mengxiao Chen, Gang Chen, Wangcai Zou, Yanqing Zhao, Qiang Zhao

**Affiliations:** School of Chemical Engineering, Sichuan University, No.24 South Section 1, Yihuan Road, Chengdu 610065, China

**Keywords:** theranostic, near-infrared quantum dots, multifunctional liposomes, magnetothermal properties, cell imaging

## Abstract

Multifunctional theranostic systems are a recent important development of medical research. We combined the characteristics of near-infrared luminescent quantum dots and thermosensitive magnetoliposomes to develop a multifunctional nano-diagnostic material. This system is based on near-infrared magnetic thermosensitive liposomes, which encapsulate drugs and can control drug localization and release. After incubating cancer cells with the liposomes, the state of the cells was analyzed in real time by near-infrared imaging. Cell viability was significantly inhibited by heat treatment or alternating magnetic field treatment, which thus improved the anti-cancer properties of the liposomes. In the future, by combining near-infrared imaging technology and an external high-frequency alternating magnetic field, we could not only detect cancer cells noninvasively but also conduct image-guided treatments for cancer.

## 1. Introduction

Cancer is known as the silent killer that affects the health of millions of people around the world [1]. Conventional chemotherapy is one of the most widely used clinical methods to improve the chances of patient survival [2,3]. As an anti-cancer drug, Paclitaxel (PTX) is one of the most effective and potent drugs used against a wide range of solid tumors, such as breast cancer, ovarian cancer, and non-small cell lung cancer [4]. However, PTX is poorly soluble in water [5]. Taxol^®^, a commercially available preparation, consists of a micellar dispersion of PTX in Cremophor EL and dehydrated ethanol (1:1 *v*/*v*). Cremophor EL can improve PTX solubility and allow its intravenous administration. However, the therapeutic effect of Taxol^®^ is associated with serious dose-limiting toxicities [6]. To overcome these disadvantages of pure PTX, the development of PTX delivery systems, including liposomes, polymeric nanoparticles, micellar dispersions, and cyclodextrin complexes, has been investigated [7]. Liposomal formulations have been suggested as potentially better options for the delivery of this drug, since they can minimize detrimental toxicities while enhancing the therapeutic efficacy of PTX [8,9,10,11].

However, drug release from traditional liposomes is slow and uncontrolled [12]. To overcome these limitations, researchers have begun to focus on stimuli-responsive liposomes. These liposomes can achieve rapid drug release under stimulation by pH [13], ultrasound [14,15], temperature [16], or enzymes [17]. Thermosensitive liposomes (TSL), which can significantly improve the release efficiency of loaded drugs once stimulated by appropriate temperatures, have received extensive attention [18]. By incorporating magnetic nanoparticles into the TSL, this new type of liposomes, called thermosensitive magnetoliposomes, could increase the release and therapeutic efficacy of the carried drug [19,20]. Pradhan et al. [21] successfully demonstrated that thermosensitive magnetoliposomes containing doxorubicin could be used for cancer hyperthermia and drug delivery. A study of thermosensitive magnetoliposomes containing 5-flurouracil and doxorubicin has also been reported [22,23].

To perform precise treatments, the integration of near-infrared fluorescence imaging and therapy into theranostic systems is an efficient strategy [24,25,26,27,28]. The wavelength range of 650–1000 nm of near-infrared fluorescence imaging is known as the first near-infrared window (NIR-I), which can achieve a tissue penetration depth of up to 10 mm [29,30,31,32]. As near-infrared imaging agents, the near-infrared dyes cyanines, phthalocyanines, and porphyrin derivatives are commonly used [33,34,35,36]. However, most of NIR dyes are characterized by poor stability, rapid decomposition in aqueous solution, low quantum yield, and lack of tumor-targeting specificity [37]. The practical applications of NIR dyes in medical diagnosis and treatment are limited [38,39]. Compared with NIR dyes, near-infrared luminescent quantum dots not only have good sensitivity and selectivity but also have a long molecular imaging period [40]. Therefore, by combining the characteristics of near-infrared quantum dots with those of drug-loaded thermosensitive magnetoliposomes, near-infrared multifunctional liposomes were developed.

**Figure 1:**
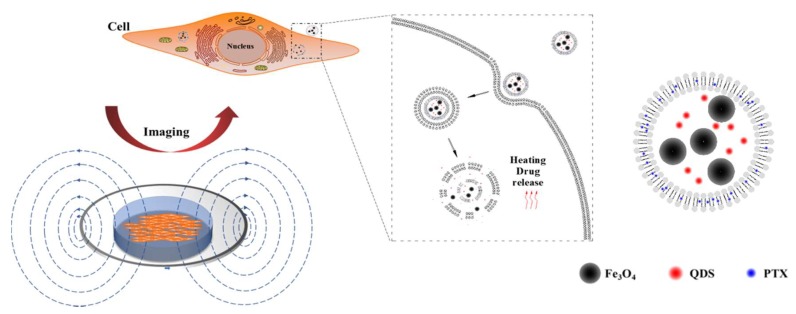
Schematic diagram of cancer cell detection and therapeutic analysis using thermosensitive liposomes (TLs) loaded with paclitaxel (PTX), near-infrared quantum dots, and magnetic nanoparticles (PTX–NMTSLs).

In this paper, thermosensitive liposomes were the basic material to prepare thermosensitive liposomes loaded with PTX, near-infrared quantum dots, and magnetic nanoparticles (PTX–NMTSLs). The response of cancer cells to PTX–NMTSLs could be observed by near-infrared fluorescence imaging. When the liposomes accumulated in the target cells, damage of the liposome membrane structure and drug release were achieved by applying an alternating magnetic field (AMF) or external heating so to generate high heat. In this way, accuracy of disease diagnosis and treatment could be improved by image guidance and drug controlled release (Figure 1).

## 2. Results and Discussion

### 2.1. Characterization of Blank Liposomes, PTX-Ls, and PTX–NMTSLs

PTX–NMTSLs were prepared by the thin-film dispersion method. The lipids of PTX–NMTSLs were composed of PTX, dipalmitoylphosphatidylcholine (DPPC), 1,2-distearol-sn-glycero-3-phosphoglycerol sodium salt (DSPG-Na), and 1,2-diacyl-sn-glycero-3-phosphoethanolamine-N-[methoxy(poly(ethyleneglycol))-2000] (DSPE–MPEG-2000). The formulation and preparation were optimized by investigating the encapsulation rate of paclitaxel. As displayed in Table S1, the encapsulation rate of paclitaxel in PTX–NMTSLs was 86.46 ± 1.43%. The iron content in PTX–NMTSLs was 116.33 ± 0.15 μg/mL, as determined by the O-phenanthroline spectrophotometric method.

The average particle size, polydispersion index (PDI), and zeta potential of blank liposomes (BLs), liposomes loaded with PTX (PTX-Ls), and PTX–NMTSLs in aqueous solution were measured by a laser particle size analyzer. As displayed in Figure S1, the diameters of BLs, PTX-Ls, and PTX–NMTSLs in aqueous solution were 57.82 ± 2.41, 67.25 ± 2.26, and 102.5 ± 2.36 nm, respectively. Compared with the other two kinds of particles, the size of PTX–NMTSLs was larger. This was because they consisted of Fe_3_O_4_ nanoparticles modified with trisodium citrate. The size of the three types of particles was less than 200 nm, which made them difficult to be engulfed by the mononuclear phagocytic system (MPS) [41,42]. The PDI values were calculated to determine the degree of dispersion of polymer micelles or particles. The PDI values of BLs, PTX-Ls, and PTX–NMTSLs were 0.228, 0.268, and 0.234, respectively. All values were less than 0.3, which suggested that these particles had a narrow size distribution. Electrostatic repulsion between particles could effectively prevent agglomeration, and their negative charge was beneficial to prolong the time liposomes were present in the blood circulation. The zeta potentials of BLs, PTX-Ls, and PTX–NMTSLs were −12.4 ± 0.4 mv, −15.1 ± 0.3 mv, and −19.8 ± 0.6 mv, respectively.

**Figure 2:**
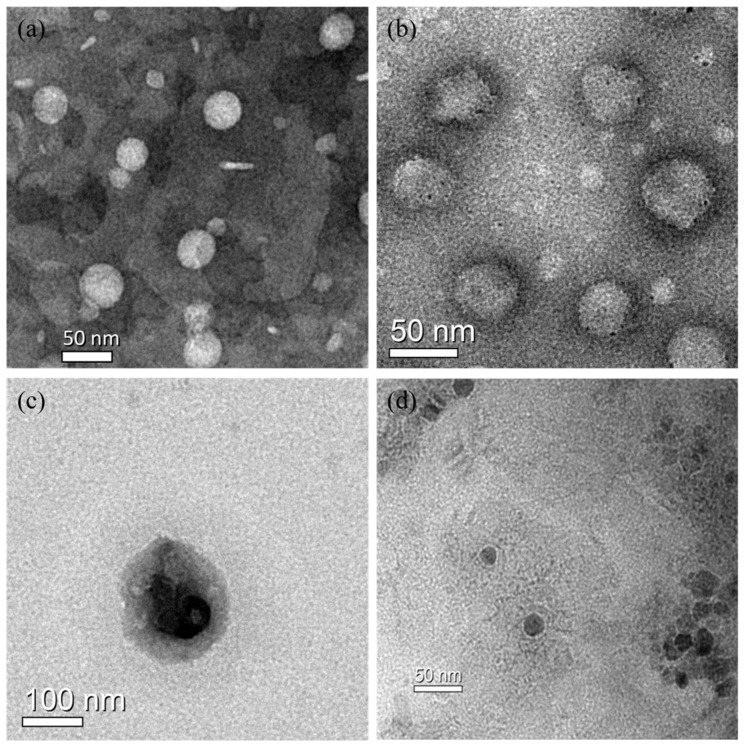
TEM images: (**a**) blank liposomes (BLs); (**b**) liposomes loaded with PTX (PTX-Ls); (**c**) PTX-NMTSLs; (**d**) Fe_3_O_4_ nanoparticles.

In order to further evaluate the diameter of the liposomes, the morphologies and sizes of BLs, PTX-Ls, and PTX–NMTSLs were observed by TEM (Figure 2a,b,c). We observed that these nanoparticles appeared as spherical vesicles. The sizes of Fe_3_O_4_ nanoparticles, BLs, PTX-Ls, and PTX–NMTSLs were about 20 nm, 50 nm, 60 nm and 100 nm, respectively. The results of the TEM analysis agreed with those of the laser particle size analyzer.

To facilitate the drug release through the application of external heat, the phase transition temperature of thermosensitive liposomes should be higher than the normal body temperature. The ratio of lipids in the liposomes was regulated to obtain adequate samples. Differential scanning calorimetry (DSC) was used for determining the phase transition temperature. The DSC thermogram (Figure S2) identified the main phase transition peaks and peak width of BLs and PTX-NMTSLs, respectively. It was found that the liposomes composed of DPPC had an obvious phase transition at 41.38 °C, which was consistent with previous published results [21,43]. The phase transition of PTX-NMTSLs was at 40.68 °C. The addition of Fe_3_O_4_, quantum dots, and drugs had a certain effect on the phase transition temperature of liposomes, but the effect was small, and the phase transition temperature remained within the required temperature range.

In order to choose the best excitation light source, the excitation spectrogram of CdSeTe quantum dots were determined by fluorescence spectrophotometry (Figure S3). The quantum dots showed the strongest absorption at 467 nm. Therefore, the wavelength of 467 nm was selected as the excitation wavelength. The fluorescence emission spectra of quantum dots and PTX–NMTSLs were obtained under the excitation of 467 nm (Figure S3b,c). The strongest emission wavelengths of quantum dots and PTX–NMTSLs were at 726 nm and 725 nm (λ_ex_ = 467 nm), respectively. This indicated that the fluorescence performance of the quantum dots was very stable when they were wrapped in liposomes.

PTX–NMTSLs were placed in an external magnetic field to verify their magnetic response. As shown in Figure S4, PTX–NMTSLs could move quickly in the direction of the magnetic field, indicated by the arrow. With the increase of time, the amount of PTX–NMTSLs increased gradually, indicating that PTX–NMTSLs had a good magnetic response.

To sum up, the prepared PTX–NMTSLs with uniform small particle size and phase transition temperature of 40.68 °C showed good magnetic response and near-infrared fluorescence performance. Such liposomes, which combine magnetic targeting and fluorescence properties, have the potential to deliver drugs to target areas, while allowing visual monitoring of the process.

### 2.2. In Vitro Stability and Release Study

**Figure 3:**
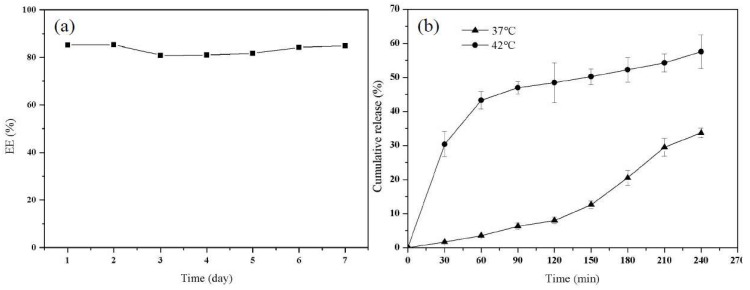
(**a**) Encapsulation efficiency of PTX–NMTSLs at different times; (**b**) temperature-dependent release curve of PTX from liposomes at 37 °C and 42 °C.

In this work, the stability of PTX–NMTSLs was evaluated by determining their encapsulation rate. The prepared PTX–NMTSLs were stored at 4 °C. As seen in Figure 3a, the encapsulation rate of the liposomes measured in a week was between 80.56% and 85.43% under the storage condition of 4 °C. The liposome solution was monitored by eye and appeared without stratification or precipitation. This indicated that the storage of the liposomes was stable in the applied conditions. To determine PTX release in vitro (Figure 3b), the cumulative release rate of PTX in PTX–NMTSLs was measured. The cumulative release rate of PTX was different at different temperatures. At 37 °C, it was about 3.57% in an hour and 33.71% in 4 h. In contrast, PTX was released rapidly within an hour at 42 °C, and the cumulative release was about 43.28%. This indicated that the prepared liposomes had a good temperature sensitivity and that the release of PTX could be controlled by adjusting the temperature and was significantly enhanced by external heat.

### 2.3. Comprehensive Effects of Near-Infrared Multifunctional Liposomes on Cancer Cells

**Figure 4:**
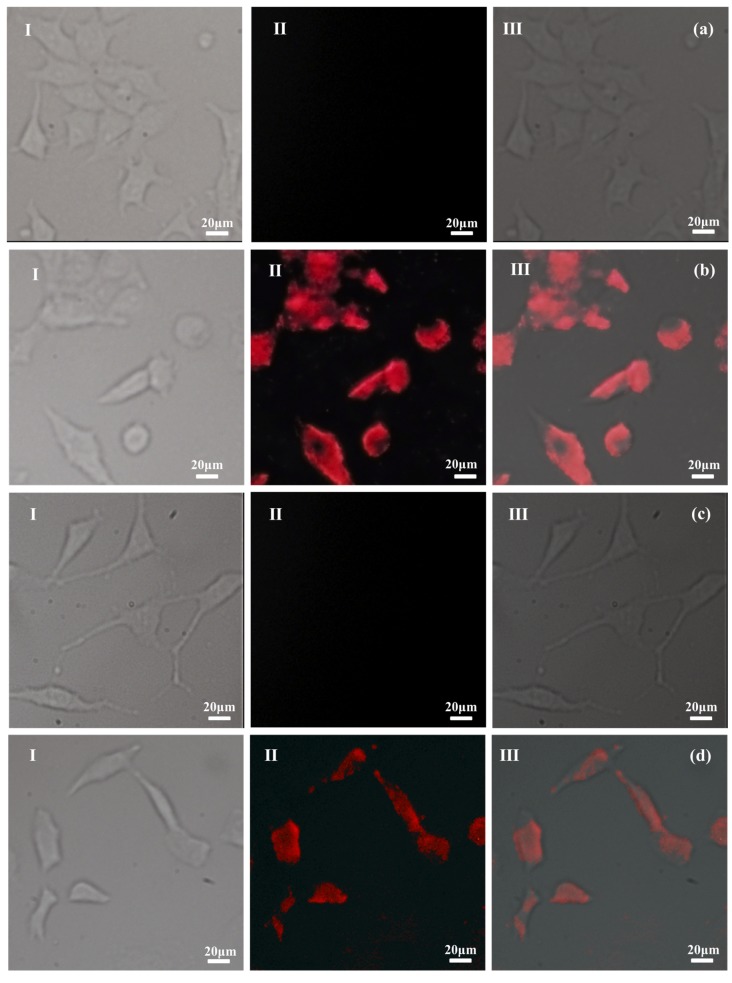
Fluorescence microphotographs: (**a**) MCF-7 cells; (**b**) MCF-7 cells after incubation with PTX-NMTSLs; (**c**) SKOV-3 cells; (**d**) SKOV-3 cells after incubation with PTX-NMTSLs. (Images in the left column (I) were obtained using an optical microscope, those in the middle column (II) by using a near-infrared (NIR) fluorescent microscope, and those in the right column (III) by overlapping the signals from the two channels).

In order to investigate the possibility to image PTX–NMTSLs by near-infrared fluorescence at the cellular level, PTX–NMTSLs were co-incubated with MCF-7 cells and SKOV-3 cells. The analysis was carried out using a fluorescence microscope equipped with a CCD camera and an analysis software. As seen in Figure 4b,d, a red fluorescence signal could be detected by the microscope, and the location of near-infrared fluorescence was consistent with the location of the cells. Meanwhile, the red signal was not detected in the control group (Figure 4a,c). This proved that PTX–NMTSLs could be successfully imaged at the cellular level using near-infrared fluorescence under 467 nm.

**Figure 5:**
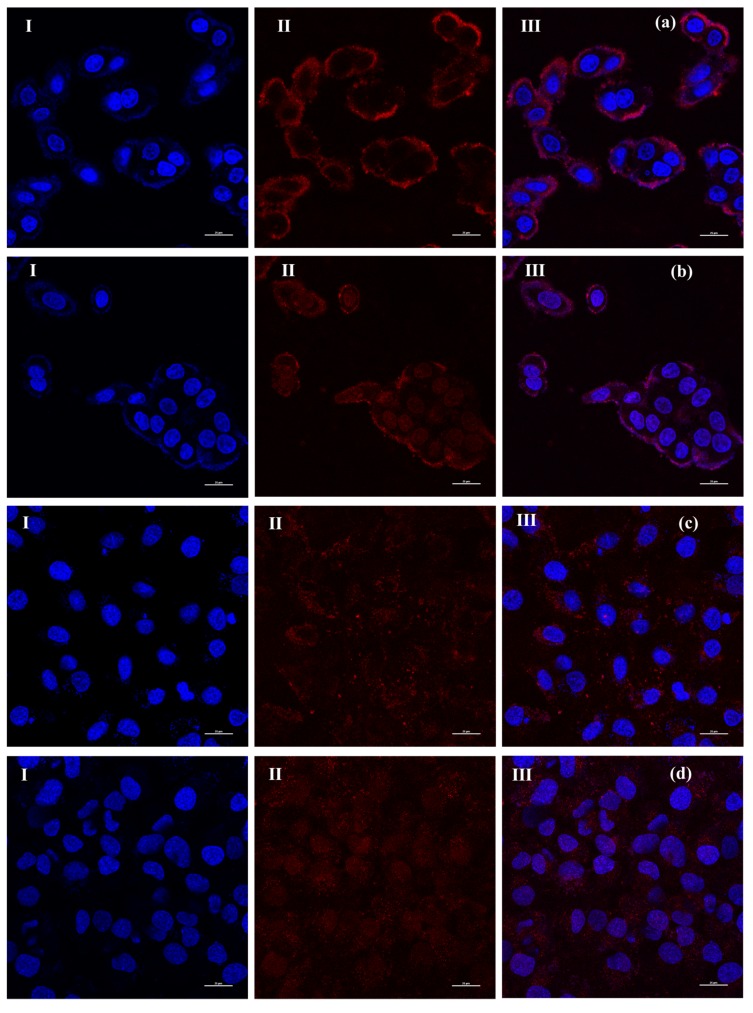
Laser scanning confocal microscopy (LSCM) images of cells after incubation with PTX-NMTSLs. (**a**) MCF-7 cells, 2 h; (**b**) MCF-7 cells, 4 h; (**c**) SKOV-3 cells, 2 h; (**d**) SKOV-3 cells, 4 h. (Cells in the left column (I) were stained with DAPI; cells in the middle column (II) were detected using the laser at the wavelength of 467 nm; images in the right column (III) were obtained by overlapping the signals from the two channels).

The drug delivery and real-time labeling capabilities of PTX–NMTSLs were further investigated. MCF-7 and SKOV-3 cells were incubated with PTX–NMTSLs for 2 h and 4 h and then analyzed by laser scanning confocal microscopy (LSCM). The interaction between PTX–NMTSLs and the cells is shown in Figure 5. After incubation for 2 h, the fluorescent images indicated that PTX–NMTSLs had gathered around the cells and entered the cytoplasm (Figure 5a,c), while after 4 h, PTX–NMTSLs had entered the nucleus (Figure 5b,d). This experiment not only showed that PTX–NMTSLs was rapidly absorbed by the cells, but also allowed following the process of cellular uptake in real time.

**Figure 6:**
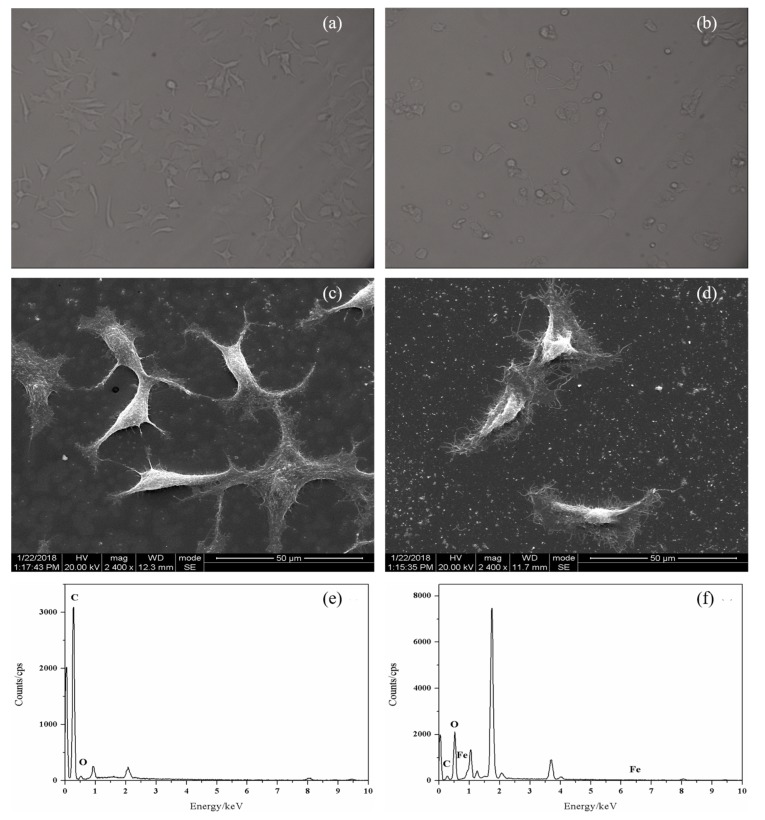
Microscopy images: (**a**) untreated MCF-7 cells; (**b**) MCF-7 cells after 48 h of co-incubation with PTX-NMTSLs. SEM images: (**c**) untreated MCF-7 cells; (**d**) MCF-7 cells after co-incubation with PTX-NMTSLs. EDS images: (**e**) untreated cells; (**f**) cells after co-incubation with PTX-NMTSLs.

To get more information about the response of cancer cells to PTX-NMTSLs, MCF-7 cells treated with PTX–NMTSLs were analyzed by scanning electron microscopy (SEM) and energy dispersive spectroscopy (EDS). As shown in Figure 6c,d, MCF-7 cells treated with PTX–NMTSLs had a rough surface and a deformed shape compared with normal cells, which were mainly due to the adhesion of PTX–NMTSLs on the cell surface and the stress response of the cells. As shown in Figure 6e,f, carbon (0.277 keV) and oxygen (0.525 keV), important elements of cell membranes, were found on the cell surface. Iron (0.615 keV, 0.703 keV, 6.404 keV, 7.058 keV) was only detected on the surface of treated cells. The elemental analysis revealed that Fe element originated from PTX–NMTSLs. These results further confirmed that PTX–NMTSLs, with their good loading and drug releasing capacities, have significant research and application potential.

**Figure 7:**
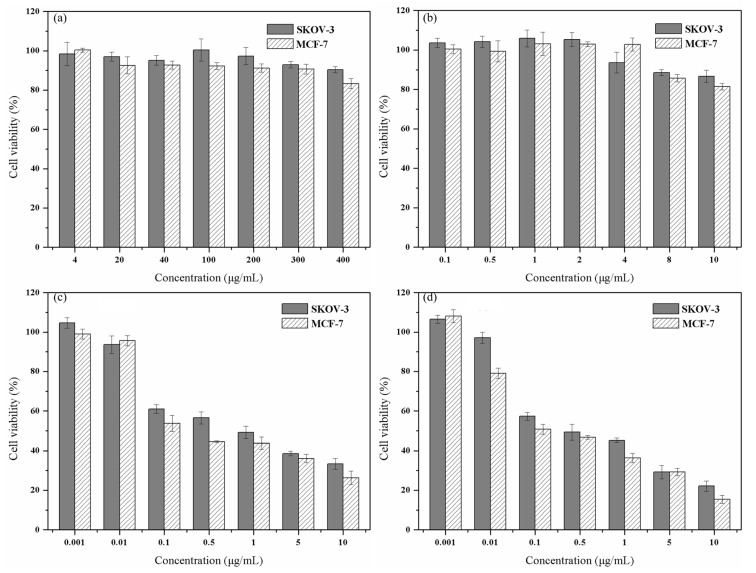
(**a**) Cell viability of SKOV-3 and MCF-7 cells cultured with BLs for 48 h. (**b**) Cell viability of SKOV-3 and MCF-7 cells cultured with magnetic liposomes (MLs) for 48 h. Cell viability of SKOV-3 and MCF-7 cells cultured with PTX-NFTSLs under different treatments: (**c**) alternating magnetic field (AMF); (**d**) 42 °C water bath.

To verify the anticancer ability of PTX-NMTSLs, we used the MTT assay. As shown in Figure 7a,b, when the concentration of blank liposomes (BLs) or magnetic liposomes (MLs) reached a high value, the survival rates of MCF-7 and SKOV-3 cells were still greater than 80%. These results indicated that BLs and MLs had low cytotoxicity and good biocompatibility.

After co-incubation with PTX-NMTSLs, the cancer cells were treated with an alternating magnetic field or by heating in a 42 °C water bath. When the concentration of PTX was greater than 0.01 μg/mL, the viability of MCF-7 and SKOV-3 cells decreased. PTX–NMTSLs were lethal to both types of tumor cells within a certain concentration range. We observed a dose–effect relationship between the survival rate of the cells and the concentration of PTX. When the concentration of PTX was 10 μg/mL, the statistical distribution showed that the cell survival rates of MCF-7 and SKOV-3 cells were 33.36 ± 1.87% and 41.97 ± 3.75%, respectively. After 30 min of alternating magnetic field treatment, the survival rates of MCF-7 and SKOV-3 cells were 26.30 ± 3.44% and 33.39 ± 2.72%, respectively (Figure 7c). After an hour of water bath treatment (42 °C), the survival rates of MCF-7 and SKOV-3 cells were 15.45 ± 1.99% and 22.14 ± 2.59%, respectively. This also indicated that PTX–NMTSLs had good thermosensitivity and a magnetocaloric effect. Fe_3_O_4_ in PTX–NMTSLs generated heat in the alternating magnetic field. The accumulated heat made the liposomes reach their phase transition temperature, which destroyed their stable structure and released the drug quickly. The high concentrations of drug reached locally led to the death of the cancer cells.

## 3. Materials and Methods

### 3.1. Reagents and Chemicals

All chemicals were analytical-grade and used directly without any further purification. Chloroform (CHCl_3_) was bought from Changzheng chemical Co. Ltd. (Guangdong, China). dipalmitoylphosphatidylcholine (DPPC), DSPG-Na, and DSPE–MPEG–2000 were bought from Xi’an Ruixi Biological Technology Co. Ltd. (Xi’an, China). Paclitaxel (PTX) was bought from Wuhan Yitai Technology Co. Ltd. (Wuhan, China). Dulbecco’s modified Eagle’s medium (DMEM) and fetal bovine serum (FBS) were purchased from HyClone (Logan, UT, USA). MTT and penicillin–streptomycin were bought from Biosharp (Hefei, China). Dimethyl sulfoxide (DMSO) was obtained from Sigma-Aldrich (St.Louis, MO, USA). MCF-7 and SKOV-3 cells were obtained from the National Engineering Research Center for Biomaterials (Sichuan University). The nano-Fe_3_O_4_ magnetic fluids and the near-infrared CdSeTe quantum dots were provided by our laboratory [44,45,46].

### 3.2. Apparatus

High-resolution transmission electron microscopy (HRTEM) imaging was performed with an FEI Tecnai GF20S-TWIN equipment (FEI Ltd., Hillsboro, OR, USA). Energy-dispersive spectrometry (EDS) results were obtained using an FEI INSPECT F scanning electron microscope (FEI Ltd., Hillsboro, OR, USA). Particle sizes were determined by using a Mastersizer 2000 (Malvern Instruments Ltd., Worcestershire, UK) laser particle size analyzer. Fluorescent spectra were obtained with a F97-Pro fluorospectrophotometer (Lengguang tech. Ltd., Shanghai, China). All fluorescent microscopy images were obtained using a Shun Yu XD30-RFL instrument. Laser scanning confocal microscopy (LSCM) images were obtained with a Leica TCS SP5 equipment. Optical density (OD) was measured by a micro-plate reader (KHB ST-360, Shanghai, China).

### 3.3. Preparation of PTX–NMTSLs

PTX–NMTSLs were prepared by the thin-film dispersion method. Typically, 0.8 mL of a DPPC chloroform solution (20 mg/mL), 0.2 mL of a DSPG-Na solution (5 mg/mL), 0.4 mL of a DSPE–MPEG–2000 chloroform solution (5 mg/mL), 0.0475 mL of a PTX solution (10 mg/mL), and 0.9275 mL of chloroform were placed into a round-bottomed flask. Then, nitrogen gas was used to remove the organic solvent. After that, the flask was placed in a vacuum dryer overnight. A uniform thin film was obtained on the bottle wall at last. Then, an appropriate amount of Fe_3_O_4_ magnetic fluid, CdSeTe quantum dots, and PBS buffer solution (pH = 7.4) were added. After hydration for 60 min at 60 °C and ultrasound application for 30 min, PTX–NMTSLs were obtained. The prepared PTX–NMTSLs were filtered through a membrane (0.22 μm) and stored at 4 °C for later use.

### 3.4. In Vitro Stability and Release Study

In this paper, the stability of the liposomes was evaluated by detecting their change of encapsulation rate. The encapsulation rate of the liposomes was measured within one week at 4 °C. Each group was tested 3 times. The cumulative release rate of PTX from PTX–NMTSLs was determined in an in vitro release study. The in vitro release of PTX from a PTX–NMTSLs solution was determined using dialysis bags (MWCO 14,000 Da). PTX–NMTSLs solutions were placed into dialysis bags and immersed in 30 mL PBS (pH 7.4). Then, the test bags were put in a thermostatic oscillator at a stirring rate of 100 rpm. The temperature was controlled at 37 °C and 42 °C, respectively. At predetermined time points, 3 mL of media was taken out of the bags for testing. To maintain the same volume, the system was supplemented with 3 mL of fresh medium. The sample solution was centrifuged at 10,000 rpm for 10 min, and the concentration of PTX in the supernatant was then detected.

### 3.5. Comprehensive Effects of Near-Infrared Multifunctional Liposomes on Cancer Cells

SKOV-3 and MCF-7 cells were cultured in α-MEM and H-DMEM media, respectively. Each medium was supplemented with 10% FBS and antibiotics (1% streptomycin and 1% penicillin). All cells were cultured at 37 °C in a humidified atmosphere with 5% CO_2_. After 24 h of cell culture, PTX–NMTSLs were added to the culture dishes, and incubation was performed for 2 h and 4 h. Then, the cells were washed with PBS (pH =7.4), fixed with 4% formaldehyde for 10 min, stained with DAPI (1 μg/mL) for 5 min, and detected using an inverted fluorescence microscope and a laser scanning confocal microscope under the excitation of 467 nm. To better analyze the response of the cancer cells co-incubated with PTX–NMTSLs, MCF-7 cells treated with PTX–NMTSLs were analyzed by scanning electron microscopy (SEM) and energy-dispersive spectroscopy (EDS).

An established colorimetric MTT assay was used for the survival analysis. MCF-7 and SKOV-3 cells were incubated with different concentrations of blank liposomes (4, 20, 40, 100, 200, 300, 400 μg/mL), MLs (Fe_3_O_4_ concentration 0.1, 0.5, 1, 2, 4, 8, 10 μg/mL), and PTX–NMTSLs (PTX concentration 0.001, 0.01, 0.1, 0.5, 1, 5, 10 μg/mL) for 48 h. The cells co-incubated with PTX–NMTSLs were divided into three groups. One group was treated with an alternating magnetic field for 30 min, one group was thermally treated by placing the cells in a water bath at 42 °C for 1 h, and the remaining group was left intreated (control). For the MTT assay, 100 μL of stock MTT (5 mg/mL) was added to each well, and incubation was carried out at 37 °C for 4 h. Then, formazan in the cells was dissolved with DMSO. Finally, the absorbance at 492 nm was tested by a micro-plate reader. All experiments were performed six times. Cell viability was calculated by the formula:

(1) $$\mathrm{Cell}\;\mathrm{viability}\left(\%\right)=\left(\frac{\mathrm{OD}\;\mathrm{value}\;\mathrm{of}\;\mathrm{experiment}\;\mathrm{group}}{\mathrm{OD}\;\mathrm{value}\;\mathrm{of}\;\mathrm{control}\;\mathrm{group}}\right)\times 100\%$$

## 4. Conclusions

In summary, we successfully prepared near-infrared multifunctional liposomes and explored their effects on cancer cells. Fluorescent cellular imaging not only showed that PTX–NMTSLs was rapidly absorbed by the cells, but also allowed following the process of cellular uptake in real time. We used the MTT assay to study the effect of multifunctional liposomes on cell viability. The results showed that BLs and MLs had low cytotoxicity, while PTX–NMTSLs had lethal effects on MCF-7 and SKOV-3 cells. The inhibition of the survival rates of tumor cells co-cultured with PTX–NFTSLs increased when the cells were treated with an AMF, which, thus, increased the anti-cancer properties of PTX–NFTSLs. Therefore, PTX–NMTSLs, multifunctional liposomes with good NIR performance, magnetic thermal response, and temperature sensitivity, have the potential not only to fluorescently label cancer cells and guide treatment, but also to inhibit cell viability through controlled drug release. This indicates that near-infrared multifunctional liposomes have great potential for the detection and treatment of cancer.

## Supplementary Materials

The following are available online. Table S1: Optimization of the formulation result. Figure S1: Size distribution and zeta potential of liposomes. Figure S2: DSC graphs of BLs (a) and PTX–NMTSLs (b). Figure S3: Fluorescence excitation spectra (a) and emission spectra (b) of CdSeTe QDs. Fluorescence emission spectra (c) and fluorescence microphotograph (d) of PTX–NMTSLs. Figure S4: Fluorescence microscopy images of PTX–NMTSLs moving toward an external magnetic field.

## Abbreviations

Paclitaxel, PTX; Thermosensitive liposomes, TSL; near-infrared, NIR; near-infrared dyes, NIR dyes; quantum dots, QDs; paclitaxel-loaded, near-infrared fluorescence magnetic thermosensitive liposomes, PTX-NMTSLs; blank liposomes, BLs; PTX-loaded liposomes, PTX-Ls; dipalmitoylphosphatidylcholine, DPPC; 1,2-distearol-sn-glycero-3-phosphoglycerol sodium salt, DSPG-Na; 1,2-diacyl-sn-glycero-3-phosphoethanolamine-*N*-[methoxy(poly(ethyleneglycol))-2000], DSPE–MPEG-2000; polydispersion index, PDI; mononuclear phagocytic system, MPS; transmission electron microscopy, TEM; differential scanning calorimetry, DSC; charge coupled device, CCD; laser scanning confocal microscope, LSCM; scanning electron microscope, SEM; energy-dispersive spectroscopy, EDS; magnetic liposomes, MLs; dimethyl sulfoxide, DMSO; phosphate buffer solution, PBS; Dulbecco’s modified Eagle’s medium, DMEM; fetal bovine serum, FBS; 3-(4,5-dimethyl-2-thiazolyl)-2,5-diphenyl-2-*H*-tetrazolium bromide, MTT.
