# Supplementary material for: Comprehensive Effects of Near-Infrared Multifunctional Liposomes on Cancer Cells
Source: Molecules. 2020 Mar 1;25(5):1098. doi: 10.3390/molecules25051098 (PMC7179136; doi:10.3390/molecules25051098)
Supplement: Supplementary file 1 [file molecules-25-01098-s001.pdf]

Supporting Information

Comprehensive Effects of Near-Infrared Multifunctional Liposomes on Cancer Cells

Yiqing Deng [1], Huaying Huang [1], Mengxiao Chen [1], Gang Chen [1], Wangcai Zou [1], Yanqing Zhao [1] and Qiang Zhao [1,*]

[1] School of Chemical Engineering, Sichuan University, No.24 South Section 1, Yihuan Road, Chengdu, China, 610065; dengyq24@outlook.com (Y.D.); amindahhy@163.com (H.H.); m15198138109_1@163.com (M.C.); 13281056708@163.com (G.C.); zouwangcai1234@163.com (W.Z.); qin19940589@163.com (Y.Z.); Tel.: 0086-15184325181

* Correspondence: zhaoqiang@scu.edu.cn (Q.Z.)

1. The optimization formulation result

Table S1. Optimization of the formulation result

Factor	Result
DPPC: DSPEG-Na ratio	8:0.5
Phospholipid concentration	8 mg/mL
Hydration temperature	60 ^{0}C
Hydration time	60 minutes
Ultrasonic time	30 minutes
Phospholipid and drug ratio	40:1
The average encapsulation efficiency	86.46 ± 1.43%

2. Characterization of BLs, PTX-Ls and PTX-NMTSLs

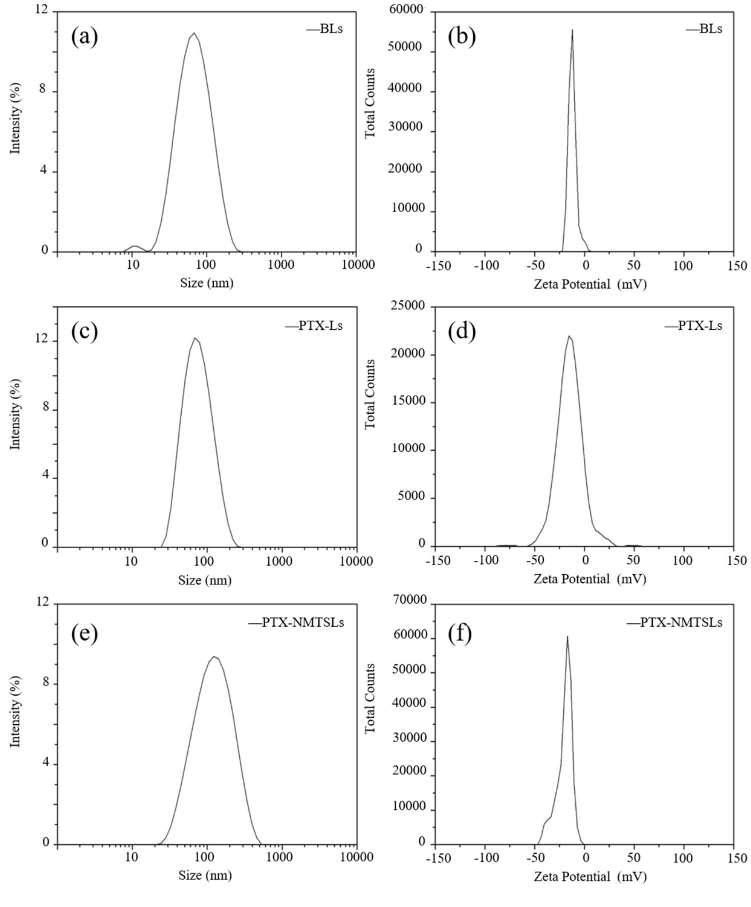

Figure S1. Size distribution and zeta potential of liposomes. (a), (c), (e) were the size distribution of BLs, PTX-Ls, PTX-NMTSLs respectively. (b), (d), (f) were the zeta potential of BLs, PTX-Ls, PTX-NMTSLs respectively.

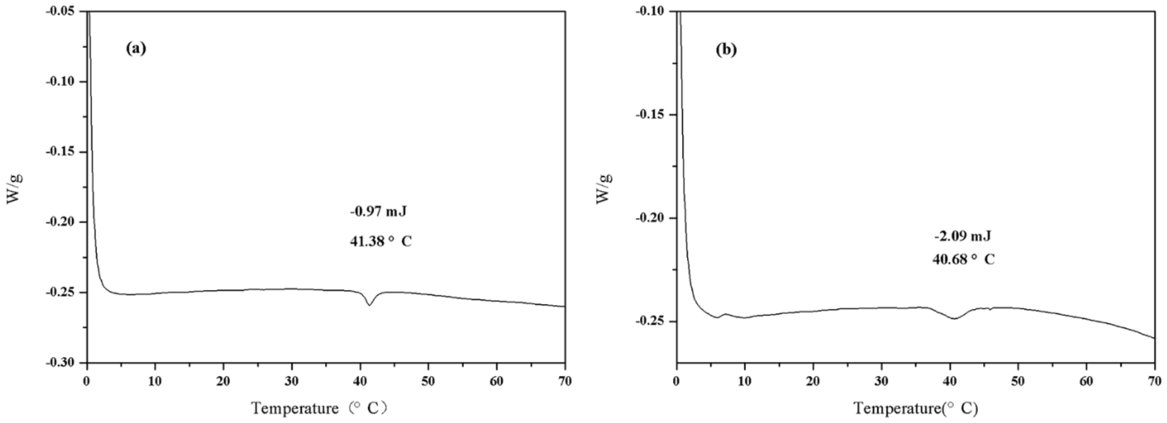

Figure S2. DSC graphs of BLs (a) and PTX–NMTSLs (b).

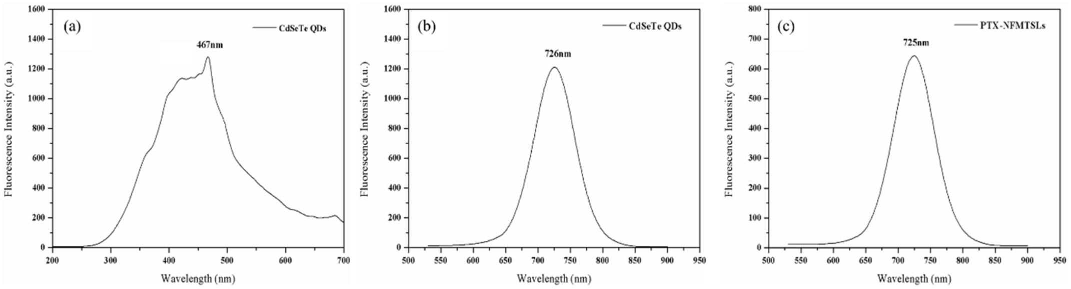

Figure S3. Fluorescence excitation spectra (a) and emission spectra (b) of CdSeTe QDs. Fluorescence emission spectra (c) and fluorescence microphotograph (d) of PTX–NMTSLs.

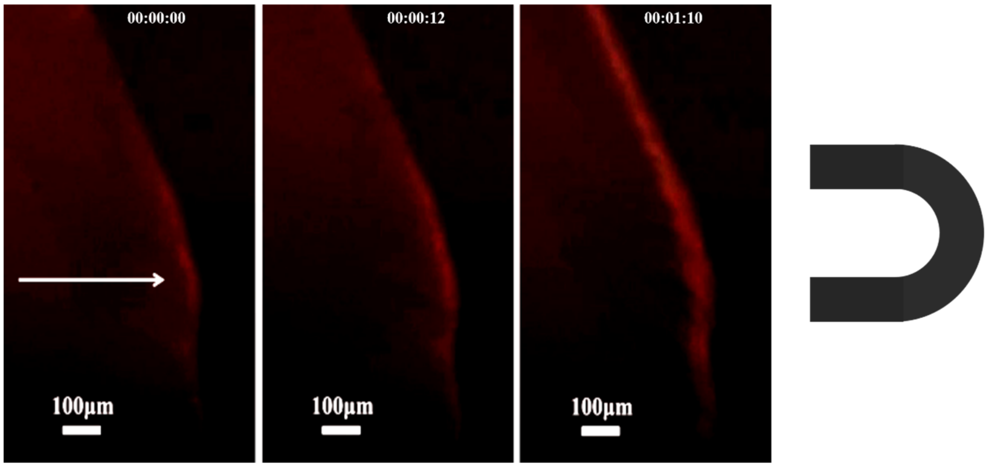

Figure S4. Fluorescence microscopy images of PTX–NMTSLs moving toward an external magnetic field.
